# Effect of Y_2_O_3_ Content on the Microstructure and Thermal Shock Resistance of Al_2_O_3_–Y_2_O_3_ Composite Coatings

**DOI:** 10.3390/ma19112381

**Published:** 2026-06-03

**Authors:** Zhipeng Hu, Li Feng, Yanchun Zhao, Zhiyuan Wei, Bingbing Liu, Chao Ma, Bo Cheng

**Affiliations:** 1 School of Materials Science and Engineering, Lanzhou University of Technology, Lanzhou 730050, China; 18294107436@163.com (Z.H.); yanchun_zhao@163.com (Y.Z.); machaccc@yeah.net (C.M.); 2 State Key Laboratory of Advanced Processing and Recycling of Nonferrous Metals, Lanzhou University of Technology, Lanzhou 730050, China; 3 School of Automotive Materials, Hubei University of Automotive Technology, Shiyan 442002, China; 20240044@huat.edu.cn (Z.W.); l18062304823@163.com (B.L.); 4 State Key Laboratory of Solar Power Generation Systems, Jiuquan Vocational Technical University, Jiuquan 735000, China

**Keywords:** Al_2_O_3_–Y_2_O_3_ composite coatings, atmospheric plasma spraying (APS), microstructural evolution, thermal shock resistance

## Abstract

Thermal shock resistance is a critical parameter for evaluating the long-term service reliability of protective coatings in high-temperature molten-salt environments. In this study, Al_2_O_3_–Y_2_O_3_ composite coatings containing 0, 2, 5, and 8 wt.% Y_2_O_3_ were fabricated on 316L stainless-steel substrates by atmospheric plasma spraying (APS). Their phase constitution, microstructure, mechanical properties, and thermal shock resistance were systematically investigated. The results showed that, with increasing Y_2_O_3_ content, the relative content of α-Al_2_O_3_ gradually increased, whereas the coating densification, microhardness, and fracture toughness first increased and then decreased. After 200 thermal shock cycles, the thermal shock resistance of the Al_2_O_3_–Y_2_O_3_ composite coatings followed the order of 5 wt.% Y_2_O_3_ > 2 wt.% Y_2_O_3_ > 8 wt.% Y_2_O_3_ > 0 wt.% Y_2_O_3_, indicating that the addition of an appropriate amount of Y_2_O_3_ significantly improves the thermal shock resistance of the coatings. Analysis of the failure mechanism further revealed that the addition of an appropriate amount of Y_2_O_3_ enhanced phase stability and optimized the coating microstructure, thereby improving the crack-propagation resistance and ultimately enhancing the thermal shock resistance. In contrast, excessive Y_2_O_3_ weakens this beneficial effect because of increased microstructural heterogeneity and a higher defect density.

## 1. Introduction

Alumina (Al_2_O_3_) ceramics possess high hardness, excellent chemical inertness, and strong barrier capability against corrosive media, and have therefore been widely used in various engineering fields [1,2,3]. Al_2_O_3_ coatings fabricated by atmospheric plasma spraying (APS) exhibit considerable potential for corrosion protection in high-temperature chloride molten-salt environments [4,5,6,7]. However, APS-fabricated Al_2_O_3_ coatings are inherently characterized by a lamellar structure formed by the rapid deposition and solidification of molten droplets, and therefore inevitably contain defects such as pores and microcracks. These defects not only degrade the toughness and load-bearing capacity of the coatings but also serve as preferential sites for stress concentration and crack propagation during thermal shock cycling, ultimately leading to premature cracking and even spallation [8,9,10,11]. Recent studies have further shown that APS coatings in complex service environments are usually not subjected to a single degradation factor, but instead experience the coupled effects of corrosion, wear, thermal shock, and thermal stresses. Rapid heating and cooling can generate thermal stresses and promote microcrack formation and interlamellar bonding degradation within the coatings, thereby affecting their structural integrity and long-term protective performance [12,13]. Therefore, for APS Al_2_O_3_ protective coatings intended for use in high-temperature chloride salt environments, thermal shock resistance is a critical criterion for evaluating their long-term service reliability. To improve the microstructural characteristics and mechanical properties of Al_2_O_3_ coatings, the addition of a second phase has been considered as an effective strategy. Previous studies have shown that second-phase additives such as TiO_2_, Cr_2_O_3_, and Y_2_O_3_ can improve the microstructure and mechanical properties of Al_2_O_3_ coatings to varying extents [14,15,16,17,18]. Among these additives, Y_2_O_3_ has gradually emerged as an important modifying constituent for Al_2_O_3_ coatings because of its high chemical stability, excellent thermal stability, and favorable thermal expansion compatibility with Al_2_O_3_. In recent years, Y_2_O_3_-containing ceramic coatings have also been widely used for surface protection of high-temperature alloys and in complex corrosive environments. Adomniței et al. [12] investigated the electrochemical corrosion behavior of YSZ coatings with high Y_2_O_3_ content, demonstrating that the phase stability, microstructure, and protective performance of Y_2_O_3_-stabilized ceramic coatings are closely related. Rong et al. [19] prepared both Al_2_O_3_ coatings and Al_2_O_3_–Y_2_O_3_ composite coatings by APS. Their results showed that the relative content of α-Al_2_O_3_ in the Al_2_O_3_–Y_2_O_3_ composite coating was higher than that in the pure Al_2_O_3_ coating, indicating that the addition of Y_2_O_3_ is beneficial for stabilizing the α-Al_2_O_3_ phase. In addition, compared with pure Al_2_O_3_ coatings, Al_2_O_3_–Y_2_O_3_ composite coatings exhibited a more stable coefficient of friction against graphite and superior wear resistance. Ma et al. [20] likewise prepared Al_2_O_3_–Y_2_O_3_ composite coatings by APS. Their study demonstrated that the overall porosity of the Al_2_O_3_–Y_2_O_3_ composite coatings was lower than that of the pure Al_2_O_3_ coating. However, with a further addition of Y_2_O_3_ content, the coating porosity increased, and the hardness decreased, whereas the fracture toughness improved. These findings indicate that the modifying effect of Y_2_O_3_ on Al_2_O_3_-based composite coatings does not increase in a simple linear manner, but is strongly dependent on the compositional ratio. Different Y_2_O_3_ contents can alter the pore structure and microstructural homogeneity of the coatings, thereby leading to differences in coating performance [21].

Although the introduction of Y_2_O_3_ has been demonstrated to regulate the microstructure and overall performance of Al_2_O_3_-based composite coatings, existing studies have mainly focused on mechanical properties and wear behavior. However, for APS-fabricated Al_2_O_3_–Y_2_O_3_ composite coatings intended for high-temperature chloride molten-salt environments, corrosion resistance alone is insufficient; the coatings must also maintain structural integrity and interfacial stability. Therefore, it remains necessary to clarify how Y_2_O_3_ content regulates the initial defect structure and how these defect-structure changes influence crack-propagation resistance and thermal cycling failure mechanisms. In view of this, Al_2_O_3_–Y_2_O_3_ composite coatings containing 0, 2, 5, and 8 wt.% Y_2_O_3_ were deposited on 316L stainless-steel substrates by APS. Combined with water-quenching thermal cycling tests conducted at 600 °C, the effects of Y_2_O_3_ content on the initial phase constitution, defect structure, mechanical properties, and thermal cycling failure behavior of the coatings were systematically investigated. This study aims to guide the optimized design of Al_2_O_3_-based protective coatings intended for service in high-temperature chloride salt environments.

## 2. Materials and Methods

### 2.1. Coating Preparation

**Figure 1:**
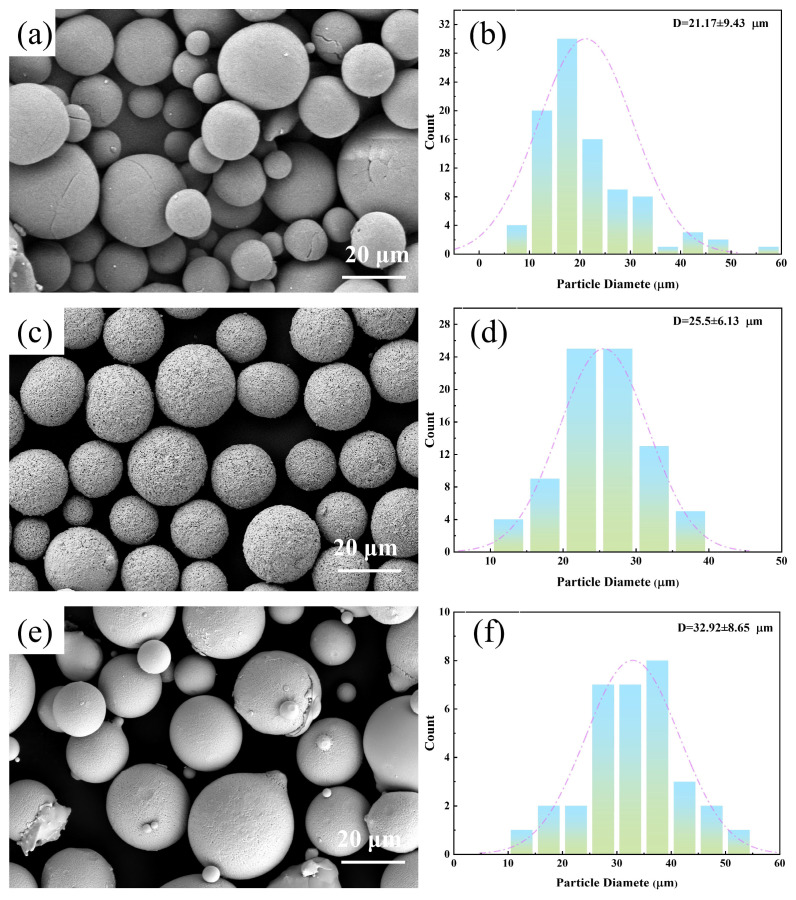
Morphologies and particle size distributions of the Al_2_O_3_, Y_2_O_3_, and NiCrAlY powders: (**a**,**b**) SEM micrograph and particle-size distribution of Al_2_O_3_ powder; (**c**,**d**) SEM micrograph and particle-size distribution of Y_2_O_3_ powder; (**e**,**f**) SEM micrograph and particle-size distribution of NiCrAlY powder.

The micron-sized Al_2_O_3_ powder (21.17 ± 9.43 μm), Y_2_O_3_ powder (25.17 ± 6.13 μm), and NiCrAlY powder (32.9 ± 8.65 μm), all supplied by Hunan Zhaoyi Thermal Spraying Material Co., Ltd. (Yiyang, China), are shown in Figure 1. Y_2_O_3_ powder was mechanically mixed with the Al_2_O_3_ powder at mass fractions of 0, 2, 5, and 8 wt.%. The powder mixtures were then ball-milled in a planetary ball mill (YXQM-4L, Hunan Changsha Mitr Instrument Equipment Co., Ltd., Changsha, China) at 100 rpm for 24 h to obtain Al_2_O_3_–Y_2_O_3_ feedstock powders with different Y_2_O_3_ contents.

Coatings were deposited on 316L stainless-steel substrates (Ø20 mm × 3 mm) by atmospheric plasma spraying (APS). Before spraying, the substrates were ultrasonically cleaned successively in acetone and ethanol for 15 min each; they were then grit-blasted with alumina particles of 50 μm in size at a pressure of 0.6 MPa to enhance the bonding strength between the substrate and the coating. A Sulzer-Metco F4 plasma spraying system was subsequently employed to sequentially deposit a NiCrAlY bond coat with a thickness of 50 ± 10 μm and a ceramic top coat with a thickness of 250 ± 10 μm. The detailed spraying parameters are listed in Table 1. For convenience, the Al_2_O_3_–Y_2_O_3_ composite coatings containing 0, 2, 5, and 8 wt.% Y_2_O_3_ were designated A0Y, A2Y, A5Y, and A8Y, respectively.

### 2.2. Thermal Shock Cycling Test

The thermal cycling test was performed using a water-quenching method. The detailed procedure was as follows. The as-prepared specimens (Ø20 mm × 3 mm) were first ultrasonically cleaned in ethanol, thoroughly dried at 120 °C, and then cooled to room temperature. The initial mass of each specimen was measured using an analytical balance and recorded as M_0_, and the surface morphology before thermal cycling was photographed. A box-type resistance furnace (KSL-1400X, Hefei Kejing Materials Technology Co., Ltd., Hefei, China) was preheated at 900 °C for 2 h and then cooled to 600 °C for use. The coated specimens were rapidly placed into the furnace and held for 10 min, after which they were immediately removed and quenched in water at 20 ± 5 °C for 20 s. The specimens were then dried with a hair dryer, weighed again, and recorded as M*_i_*, and photographed again to document the surface condition after thermal cycling. One complete thermal cycle was thus completed. This procedure was repeated until the total number of cycles reached 200.

Based on the surface photographs taken before and after thermal cycling, the spalled area was quantified using ImageJ 1.53k software, and the spallation area ratio was calculated according to Equation (1):

(1) $$P=\frac{\left(S-A\right)}{S}\times 100\%$$

where *P* is the spallation area ratio after thermal cycling (%), *A* is the residual coating area after thermal cycling (cm^2^), and S is the original surface area of the coating before thermal cycling (cm^2^).

Meanwhile, the mass loss per unit area was calculated using Equation (2):

(2) $$\frac{\Delta m}{S}=\frac{{(M}_{0}-M_{i})}{S}\;(i=1,2,3,\ldots \ldots ,200)$$

where $\frac{\Delta m}{S}$ is the mass loss per unit area of the specimen after thermal cycling (mg·cm^−2^), *M*_0_ is the mass of the specimen before thermal cycling (mg), *M_i_* is the mass of the specimen after thermal cycling (mg), and S is the original surface area of the coating before thermal cycling (cm^2^).

### 2.3. Characterization

The phase composition of the coating samples before and after thermal cycling was characterized by X-ray diffraction (XRD, D/MAX2500PC, Rigaku, The Woodlands, TX, USA). The surface and cross-sectional microstructures of the coatings were examined by scanning electron microscopy (SEM, JSM-IT500, JEOL, Tokyo, Japan) equipped with an energy-dispersive spectroscopy (EDS) system for elemental analysis. Based on the cross-sectional SEM images, 10 representative images at a magnification of 300× were selected for porosity analysis before and after thermal cycling. The average value was taken as the final porosity [22]. The three-dimensional surface morphology of the coatings was characterized using a white-light interferometer (SuperView W3, Zhongtu Instrument Co., Ltd., Shenzhen, China), and the surface roughness parameter S_a_ was obtained. For each sample, three different regions were measured, and the average value was reported. The microhardness of the coating was measured using a microhardness tester (Wilson VH1102, Buehler, Lake Bluff, IL, USA) equipped with a standard Vickers diamond indenter. Before testing, the coating samples were mounted using a mounting press (ZXQ-5H, Laizhou Huayin Testing Instrument Co., Ltd., Laizhou, China), with the coating cross-section serving as the test surface. A load of 4.9 N was applied for 15 s, and each sample was measured 15 times. The average value was then calculated to obtain the average microhardness value. To further evaluate the indentation fracture toughness of the coatings, the indentation fracture toughness was calculated using the Evans model [23]:

(3) $$K_{IC}=0.79\frac{P}{\sqrt{a^{3}}}\log\frac{4.5a}{c}$$

where *P* is the applied Vickers indentation load (N), *a* is the half-diagonal length of the indentation (μm), and c is the crack length (μm), with *c* = *a* + L, where L is the distance from the crack tip to the indentation boundary. In this study, the ratio of *c*/*a* satisfied 0.6 < *c*/*a* < 4.5. The indentation fracture toughness test was measured under a load of 4.9 N, and 15 indentation sites were selected on the cross-section of each sample for measurement. Grubbs’ criterion was then employed to identify outliers at a significance level of α = 0.05. After excluding abnormal values, eight valid datasets were retained for the calculation of the mean value and standard deviation.

## 3. Results and Discussion

### 3.1. Coatings Phase Constitution and Microstructure

**Figure 2:**
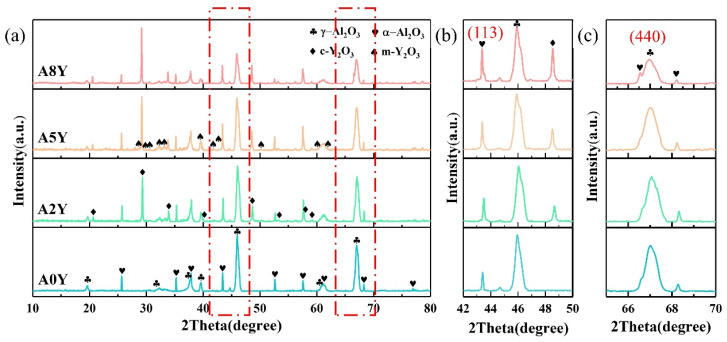
XRD patterns of Al2O3–Y2O3 composite coatings with different Y2O3 contents: (**a**) full-range patterns; (**b**) enlarged view in the 2θ range of 42°–50°; (**c**) enlarged view in the 2θ range of 65°–70°.

Figure 2 shows the XRD patterns of the Al_2_O_3_ coating and the Al_2_O_3_–Y_2_O_3_ composite coatings. No reaction phases, such as YAG, are observed in the Al_2_O_3_–Y_2_O_3_ composite coatings. Owing to the detection limit of XRD, however, the possible presence of trace reaction products or poorly crystallized nanoscale phases cannot be completely excluded. During the atmospheric plasma spraying process, Al_2_O_3_ and Y_2_O_3_ particles are co-heated and deposited in the plasma jet. However, because the durations of particle flight, splat spreading, and solidification are extremely short, the elemental diffusion and reaction between the two constituents are limited, making it difficult to form reaction products detectable by XRD. Therefore, Al_2_O_3_ and Y_2_O_3_ in the coatings largely retain their individual crystal structures, which is consistent with previous reports [19,24]. The Al_2_O_3_–Y_2_O_3_ composite coatings mainly consist of α-Al_2_O_3_, γ-Al_2_O_3_, c-Y_2_O_3_, and a small amount of m-Y_2_O_3_. In addition, m-Y_2_O_3_ is detected only as weak diffraction peaks in the A5Y and A8Y samples. To further clarify the effect of Y_2_O_3_ content on the relative proportions of α-Al_2_O_3_ and γ-Al_2_O_3_ in the coatings, a semi-quantitative analysis of the Al_2_O_3_ phase constitution was performed using the intensity ratio of the α-Al_2_O_3_ (113) and γ-Al_2_O_3_ (440) characteristic peaks, namely, I(113)/I(440). As listed in Table 2, this ratio increases progressively from 0.31 for A0Y to 0.42, 0.65, and 0.86 for A2Y, A5Y, and A8Y, respectively, indicating that the relative content of α-Al_2_O_3_ in the coatings continuously increases with increasing Y_2_O_3_ content. This trend is consistent with the results reported by Rong et al. [17]. From the viewpoint of classical heterogeneous nucleation theory, the formation of α-Al_2_O_3_ during APS rapid solidification is governed by both thermodynamic driving force and interfacial energy barrier. During plasma spraying, rapid cooling of molten Al_2_O_3_ droplets generally favors the formation of metastable γ-Al_2_O_3_. The introduced Y_2_O_3_ particles or Y_2_O_3_-rich regions may serve as thermally stable heterogeneous nucleation sites for α-Al_2_O_3_, reducing the effective nucleation barrier when favorable interfacial compatibility exists. Therefore, the intensity ratio of α-Al_2_O_3_ to γ-Al_2_O_3_ increases with increasing Y_2_O_3_ content. However, it should be noted that this peak-intensity ratio provides only a semi-quantitative comparison of the relative variation in α-Al_2_O_3_ and γ-Al_2_O_3_, rather than an absolute phase fraction. The calculated ratio may be affected by preferred orientation, peak overlap, differences in crystallinity, grain size, and residual stress. Nevertheless, because all coatings were prepared using similar APS procedures and tested under identical XRD conditions, the intensity ratio can still provide a reasonable basis for comparing the relative change in Al_2_O_3_ phase constitution among the different coatings.

**Figure 3:**
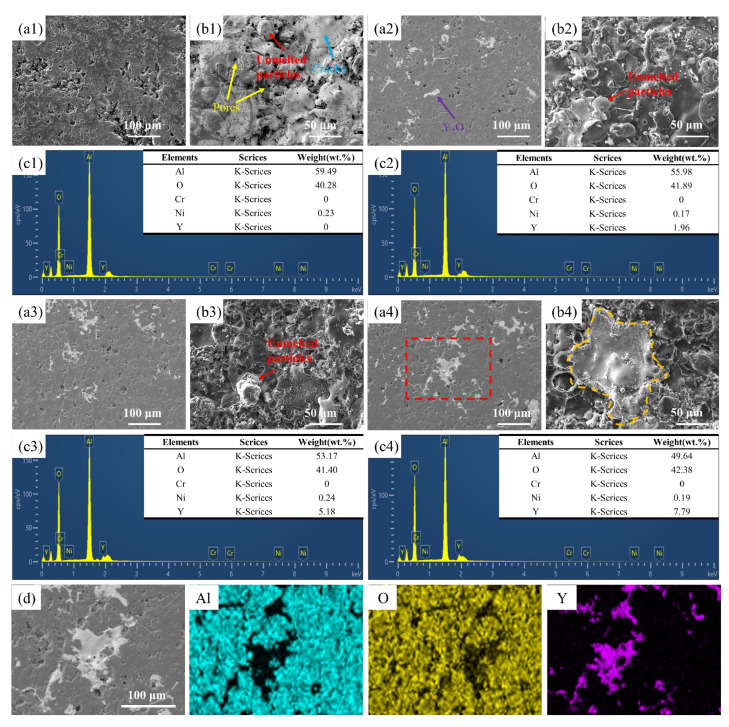
Surface morphologies and EDS analysis results of the A0Y, A2Y, A5Y, and A8Y coatings: (**a1**–**a4**) polished surface morphologies; (**b1**–**b4**) as-sprayed surface morphologies; (**c1**–**c4**) surface EDS spectra; (**d**) EDS elemental mapping results of the red-marked region in (**a4**).

**Figure 4:**
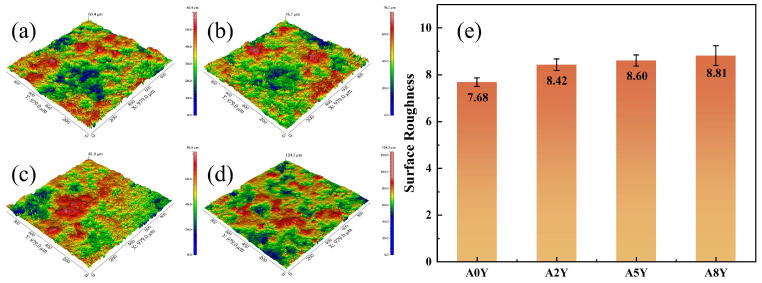
Three-dimensional surface morphologies and surface roughness of the A0Y, A2Y, A5Y, and A8Y composite coatings: (**a**–**d**) three-dimensional surface morphologies; (**e**) surface roughness values.

Figure 3 and Figure 4 show the surface morphology of the Al_2_O_3_–Y_2_O_3_ composite coatings. As shown by the polished surface morphologies in Figure 3(a1–a4), the surface of the A0Y is mainly characterized by a single dark phase, whereas randomly distributed bright regions are additionally observed in the A2Y, A5Y, and A8Y samples. According to the EDS elemental mapping results in Figure 3d, the dark phase is identified as Al_2_O_3_, while the relatively bright regions correspond to Y_2_O_3_. As further shown in Figure 3(c2–c4), the Y_2_O_3_ contents in the ceramic top coats of the A2Y, A5Y, and A8Y are 1.96, 5.18, and 7.79 wt.%, respectively. These values are in good agreement with the designed compositions, with deviations controlled within 3%, indicating that effective Y_2_O_3_ addition is achieved during the spraying process. Figure 3(b1–b4) presents the as-sprayed surface morphologies. All coatings exhibit a typical APS lamellar architecture, in which molten or semi-molten particles spread on the substrate surface to form irregular flattened splats, accompanied by a certain number of microcracks (blue arrows), pores (yellow arrows), and unmelted particles (red arrows). To further quantify the surface characteristics of the coatings, white-light interferometry was employed to characterize the three-dimensional surface topography and roughness. As shown in Figure 4a–d, pronounced surface undulations are observed in all four coatings. Moreover, the A2Y, A5Y, and A8Y exhibit greater surface protrusions and higher surface roughness than A0Y (Figure 4e), indicating that Y_2_O_3_ addition significantly enhances the surface waviness of the coatings. This phenomenon may be associated with the presence of a larger number of semi-molten or insufficiently melted particles on the coating surface after Y_2_O_3_ addition. Owing to their limited spreading capability upon impact, these particles tend to remain on the surface as raised features, thereby resulting in higher roughness for the composite coatings than in the pure Al_2_O_3_ coating.

**Figure 5:**
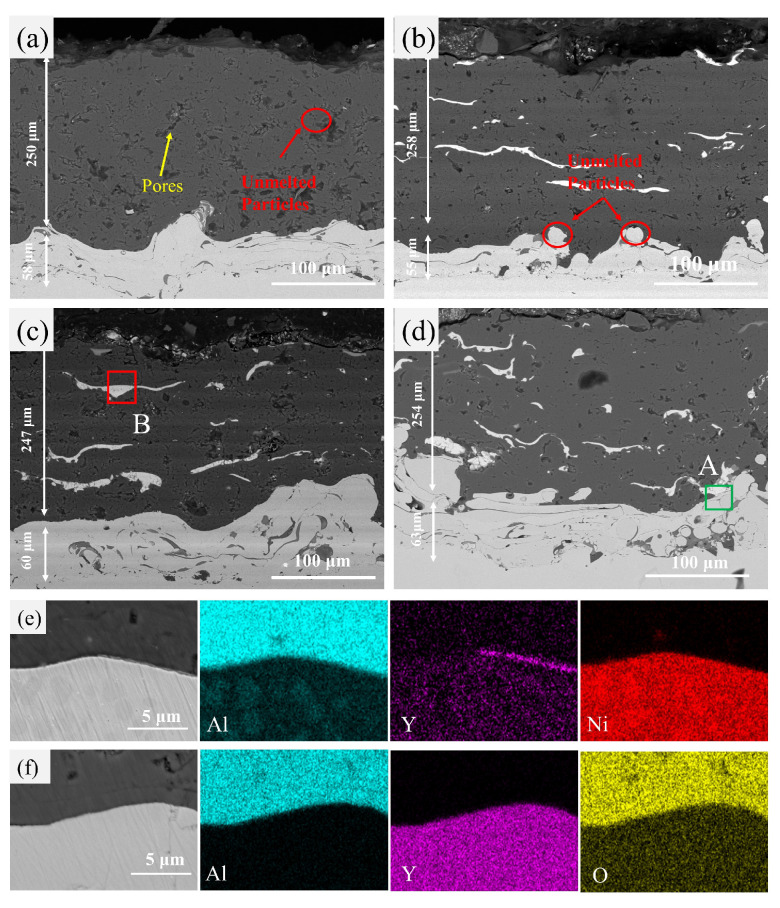
Cross-sectional morphologies and EDS analysis results of the composite coatings: (**a**–**d**) cross-sectional morphologies of the A0Y, A2Y, A5Y, and A8Y coatings; (**e**) EDS spectrum of region A marked in green; (**f**) EDS spectrum of region B marked in red.

Figure 5 shows the cross-sectional morphologies, corresponding local magnified views, and EDS results of the composite coatings. All samples exhibit a well-defined bilayer structure, in which the Al_2_O_3_-based ceramic top coat and the NiCrAlY bond coat have thicknesses of approximately 250 ± 10 μm and 50 ± 10 μm, respectively, with a continuous and distinct interface between the two layers. The EDS results from the local interfacial region (Figure 5e) show no obvious elemental transition, indicating that the bonding between the ceramic top coat and the bond coat is mainly governed by mechanical interlocking. In the Y_2_O_3_-containing samples, alternating band-like distributions of Al_2_O_3_ and Y_2_O_3_ can be observed within the ceramic top coat along the deposition direction (Figure 5b–d). In addition, the interfaces between the two phases are closely bonded and exhibit good continuity, without evident interfacial debonding or through-thickness interfacial defects, suggesting good bonding quality in the composite coatings (Figure 5f).

### 3.2. Porosity Characteristics and Mechanical Properties

**Figure 6:**
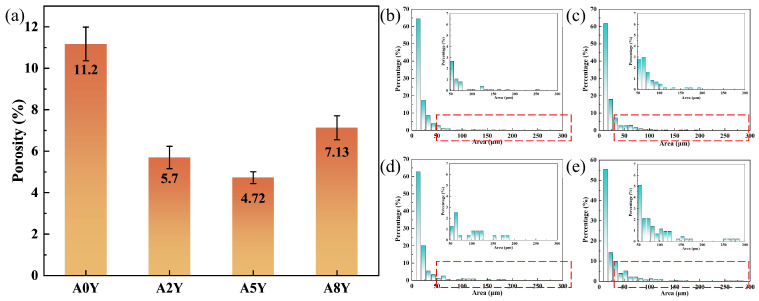
Porosity and pore size distributions of the composite coatings: (**a**) porosity of the composite coatings; (**b**–**e**) pore size distribution histograms of the A0Y, A2Y, A5Y, and A8Y coatings.

Porosity is a key microstructural factor affecting the mechanical properties and thermal shock resistance of coatings. To eliminate the influence of other variables and clarify the effect of Y_2_O_3_ content on coating porosity, the Al_2_O_3_–Y_2_O_3_ composite powders were plasma-sprayed under identical processing parameters. As shown in Figure 6, the average porosities of A0Y, A2Y, A5Y, and A8Y are 11.20 ± 0.81%, 5.7 ± 0.54%, 4.72 ± 0.28%, and 7.13 ± 0.59%, respectively. The porosity first decreases and then increases with increasing Y_2_O_3_ content, and A5Y exhibits the lowest porosity. In addition, the pores in A0Y are predominantly small, with sizes mainly distributed within 0–50 μm^2^. With increasing Y_2_O_3_ content, the proportion of small pores in A2Y and A5Y decreases markedly, whereas relatively larger pores in the range of 100–200 μm^2^ begin to appear. When the Y_2_O_3_ content is further increased to 8 wt.%, the fraction of small pores in A8Y decreases further, whereas the proportion of large pores in the range of 100–300 μm^2^ increases, corresponding to the increase in overall porosity. These results indicate that variation in the Y_2_O_3_ content not only alters the average densification level of the coatings but also affects the pore size distribution within the coatings. The increase in large pores adversely affects the mechanical properties and thermal shock resistance of the coatings. In the A8Y sample, the higher fraction of large pores and unmelted/semi-molten particles can readily act as preferential sites for local stress concentration and crack initiation. When these defects are distributed near the lamellar interfaces or the ceramic top coat/bond coat interface, they may promote the interconnection of adjacent pores, interlamellar defects, and microcracks, thereby guiding crack propagation along weakly bonded regions.

In addition, all Al_2_O_3_–Y_2_O_3_ composite coatings were prepared using the same atmospheric plasma spraying parameters, including spraying power, gas flow rate, and spraying distance. Therefore, the increased porosity of the A8Y coating was not caused by changes in spraying power, spraying distance, or jet enthalpy, but was more likely related to the differences in thermophysical properties and in-flight melting behavior between Al_2_O_3_ and Y_2_O_3_ particles under the same plasma environment. Specifically, during APS deposition of the Al_2_O_3_–Y_2_O_3_ composite coatings, the thermal and in-flight behaviors of the two types of particles differ in the plasma jet. Although the plasma flame temperature during spraying is generally higher than the melting points of both powders, the higher melting point of Y_2_O_3_ (2410 °C) compared with that of Al_2_O_3_ (2054 °C) makes Y_2_O_3_ particles more prone to insufficient melting under identical spraying conditions, thereby resulting in the retention of a certain fraction of unmelted or semi-molten particles in the coating. With increasing Y_2_O_3_ content, the number of insufficiently melted particles increases and gives rise to a shadowing effect during deposition. This hinders the filling of subsequent molten droplets and promotes the formation of larger pores, thereby increasing the overall porosity. These results indicate that the addition of an appropriate amount of Y_2_O_3_ is beneficial for reducing coating porosity and improving microstructural uniformity. However, when the Y_2_O_3_ content is further increased, although the relative content of α-Al_2_O_3_ increases, the intensified insufficient melting of the high-melting-point particles promotes the formation of large pores, thereby weakening the densification effect.

Microhardness and fracture toughness are key parameters for evaluating coating performance, as summarized in Table 3. The microhardness of the A0Y, A2Y, A5Y, and A8Y coatings shows an initial increase followed by a decrease, with A2Y exhibiting the highest value of 708.1 ± 48.4 HV_0.5_. The microhardness of plasma-sprayed coatings is closely related to both composition and microstructural characteristics. Compared with the A0Y, A2Y and A5Y exhibit lower porosity and a higher relative content of α-Al_2_O_3_, both of which enhance the local load-bearing capacity of the coatings and thereby increase the microhardness. Compared with A2Y, although A5Y still maintains a relatively low porosity, its microhardness decreases slightly. This may be associated with the increased extent of Y_2_O_3_-enriched regions, the larger number of unmelted or semi-molten particles, and the resulting variation in the effective load-bearing area. When the Y_2_O_3_ content is further increased to 8 wt.%, although the relative content of α-Al_2_O_3_ continues to increase, the coating exhibits a marked increase in unmelted or semi-molten particles and pore defects, particularly a higher fraction of large pores. This intensifies local stress concentration and weakens the overall load-bearing capacity of the coating, thereby leading to a further decrease in microhardness.

It should be emphasized that all coatings were tested under the same indentation load and analyzed using the same model. These values are mainly used to compare the relative crack-propagation resistance of coatings with different Y_2_O_3_ contents. The fracture toughness values of the Al_2_O_3_–Y_2_O_3_ composite coatings are 0.96 ± 0.25, 1.71 ± 0.31, 1.69 ± 0.40, and 1.38 ± 0.37 MPa·m^1/2,^ respectively. A2Y and A5Y exhibit comparable fracture toughness, and both show higher values than A0Y. The higher fracture toughness of the A2Y and A5Y can be mainly attributed to the following factors. First, the A2Y and A5Y contain fewer pores with smaller pore sizes, which alleviates stress concentration at pore edges and suppresses crack initiation, thereby enhancing coating toughness. In addition, an appropriate amount of Y_2_O_3_ improves interlamellar bonding and the local interfacial contact between phases, thereby increasing the energy required for cracks to propagate across lamellae. This is consistent with the findings reported by Hossein et al. [25]. When the Y_2_O_3_ content is further increased to 8 wt.%, the number of unmelted or semi-molten particles and pore defects in the coating increases markedly, accompanied by a higher fraction of large pores. This may facilitate crack interconnection, thereby reducing the crack-propagation resistance of the coating.

### 3.3. Thermal-Shock Damage Evolution and Failure Morphology

**Figure 7:**
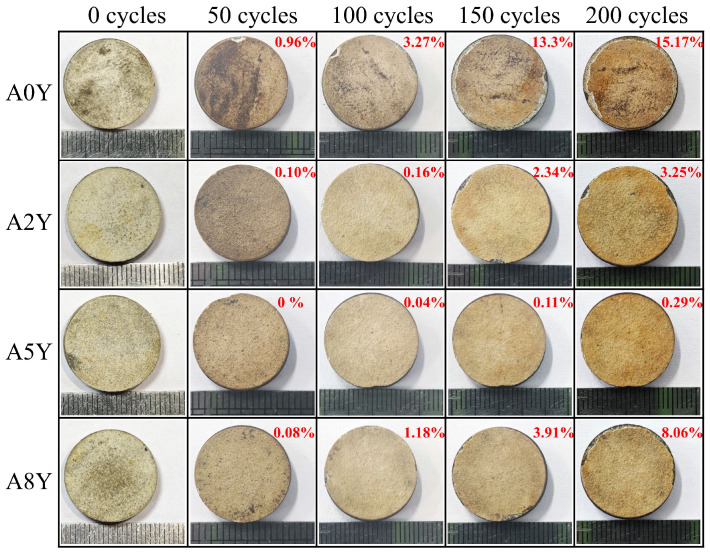
Macroscopic surface morphologies of Al_2_O_3_–Y_2_O_3_ composite coatings after different thermal shock cycles.

**Figure 8:**
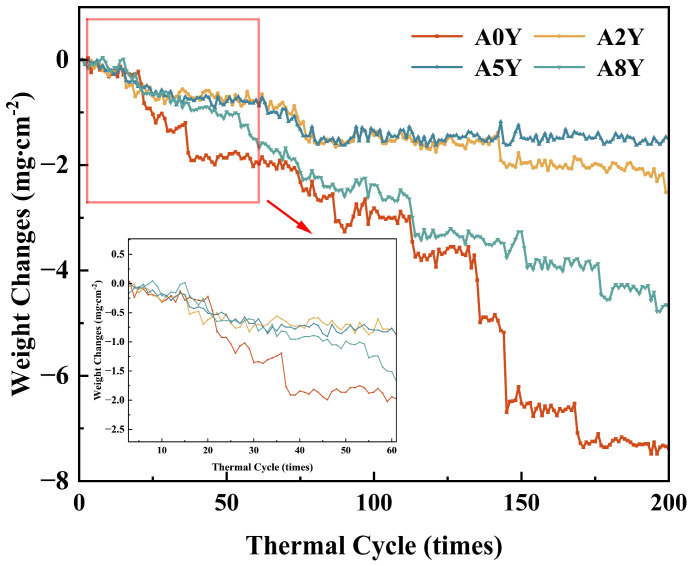
Mass loss per unit area of Al_2_O_3_–Y_2_O_3_ composite coatings during thermal shock cycling.

Figure 7 and Figure 8 present the macroscopic morphologies and mass loss per unit area of the Al_2_O_3_–Y_2_O_3_ composite coatings during water-quenching thermal cycling at 600 °C, respectively. The damage evolution of the coatings differs significantly among the samples under thermal cycling. A0Y exhibits the fastest degradation, with discernible spallation appearing on the coating surface after 50 cycles and the failure preferentially initiating at the coating edge. With increasing cycle number, the edge damage becomes progressively more severe. After 200 cycles, the spallation area ratio of the A0Y reaches 15.17%, accompanied by a mass loss of 7.5 mg∙cm^−2^. A0Y also exhibited its first pronounced mass drop after the 38th thermal shock cycle, with the mass loss reaching 2.0 mg·cm^−2^. Subsequently, abrupt mass-loss events occurred repeatedly during the 90th, 114th, 135th, and 146th cycles, with the differences in mass loss between two consecutive cycles being 0.71, 0.96, 1.29, and 1.53 mg·cm^−2^, respectively. These results indicate that the thermal-cycling damage in A0Y exhibits a distinct cumulative and accelerating trend with increasing cycle number.

In contrast, the thermal shock resistance of the coatings is markedly improved after Y_2_O_3_ addition. A2Y, A5Y, and A8Y all maintain relatively low spallation area ratios and mass losses during the first 50 cycles. The A2Y retains a low level of spallation during the 50–100 cycle stage, and its spallation area ratio increases to 3.25%, with a mass loss of 2.3 mg∙cm^−2^ after 200 cycles. Among all the samples, A5Y exhibits the best thermal shock resistance, with a spallation area ratio of only 0.29% and a mass loss of only 1.5 mg∙cm^−2^ after 200 cycles. However, when the Y_2_O_3_ content is further increased to 8 wt.%, the thermal shock resistance declines. Although A8Y remains relatively stable during the early stage, its degradation accelerates markedly after 100 cycles. Two pronounced mass-loss events occur after the 113th and 179th cycles, respectively. After 200 cycles, the mass loss of A8Y reaches 4.9 mg∙cm^−2^, and the spallation area ratio increases to 8.06%, indicating localized spallation in the middle and later stages. The spallation area ratio and the mass loss per unit area show good overall agreement, and together provide a more comprehensive characterization of damage accumulation during thermal cycling. Therefore, the thermal shock resistance of the coatings follows the order A5Y > A2Y > A8Y > A0Y.

**Figure 9:**
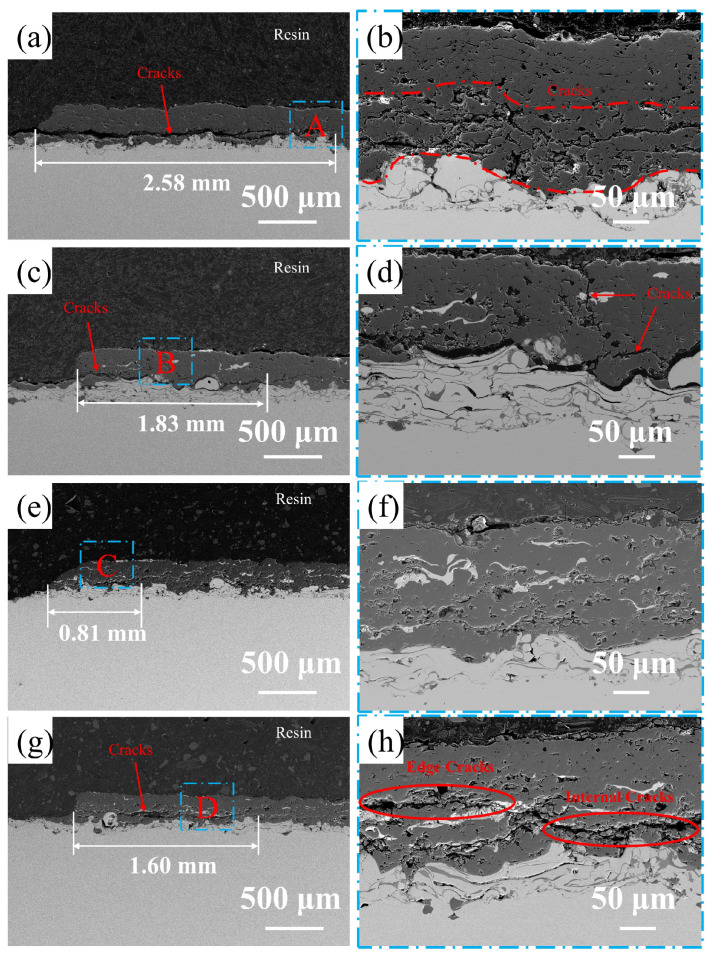
Cross-sectional SEM morphologies and corresponding local magnified views of Al_2_O_3_–Y_2_O_3_ composite coatings after 200 thermal shock cycles: (**a**,**b**) A0Y; (**c**,**d**) A2Y; (**e**,**f**) A5Y; (**g**,**h**) A8Y. The enlarged views of regions A, B, C, and D are shown in panels (**b**), (**d**), (**f**), and (**g**), respectively.

To further clarify the crack propagation paths and actual failure locations of the Al_2_O_3_–Y_2_O_3_ composite coatings during thermal cycling, the cross-sectional failure features of all samples after 200 thermal shock cycles were examined and analyzed, as shown in Figure 9. The results show that pronounced horizontal cracks are identified in the regions adjacent to the ceramic top coat/bond coat interface, and these cracks generally propagate inward from the coating edge.

Among them, A0Y exhibits the longest crack, reaching up to 2.58 mm. Moreover, a crack-dense zone with a thickness of approximately 100 μm is formed in the region adjacent to the interface, showing pronounced interlamellar cracking and local crack interconnection. In comparison, both the number and length of cracks in A2Y are significantly reduced, and no large-scale crack coalescence is observed. A5Y exhibits the best cross-sectional integrity, with only a small number of local microcracks or isolated defects observed in the ceramic top coat. With a further increase in Y_2_O_3_ content, the cross-sectional damage in A8Y becomes aggravated again, as evidenced by a marked increase in the number of cracks. Some of these cracks propagate along weakly bonded interlamellar regions and show a tendency for edge cracks to interconnect with internal cracks. These results are consistent with the observations in Figure 7 and Figure 8, namely that A0Y suffers the most severe damage, A5Y exhibits the strongest crack propagation resistance, whereas A8Y again shows aggravated damage at a higher Y_2_O_3_ content.

### 3.4. Thermal-Shock Failure Mechanism

**Figure 10:**
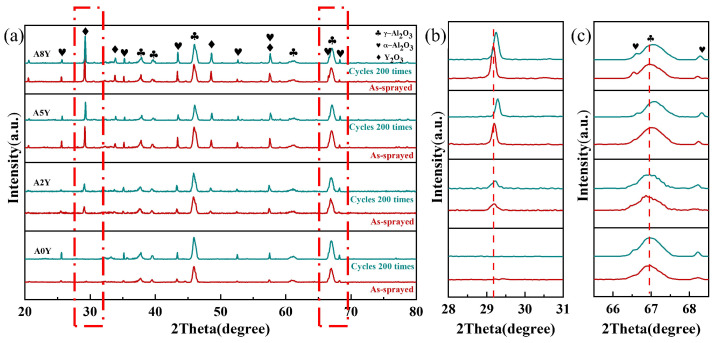
XRD patterns of the top-surface layers of Al_2_O_3_–Y_2_O_3_ composite coatings before and after thermal cycling: (**a**) full-range patterns; (**b**) enlarged view in the 2θ range of 28°–31°; (**c**) enlarged view in the 2θ range of 66°–68.5°.

Previous studies have shown that the thermal cycling lifetime of plasma-sprayed coatings is governed by multiple factors, including the thermal expansion mismatch between the ceramic layer and the metallic substrate, phase transformations in the ceramic layer, interfacial reactions between the ceramic layer and the bond coat, oxidation of the bond coat, and sintering of the ceramic layer [26,27]. Phase transformation may be an important factor leading to the top coat spallation [28]. To clarify whether any pronounced phase transformation occurs in the Al_2_O_3_–Y_2_O_3_ composite coatings during thermal cycling, the phase constitution of the failed top coats is analyzed by XRD, and the results are shown in Figure 10. After the thermal cycling test, all coatings still consist of α-Al_2_O_3_, γ-Al_2_O_3_, c-Y_2_O_3_, and a small amount of m-Y_2_O_3_, although the content of m-Y_2_O_3_ is low and is therefore not marked in the figure. Overall, no obvious changes are observed in either the positions or the types of the characteristic diffraction peaks before and after thermal cycling, indicating that 200 thermal shock cycles do not alter the principal phase constitution of the coatings. To further determine whether any local phase transformation or variation in the content of minor phases occurs during thermal cycling, the diffraction peak regions near 2θ = 29° and 2θ = 67° were analyzed in greater detail, as shown in Figure 10b,c.

After the thermal cycling test, the characteristic diffraction peaks of α-Al_2_O_3_, γ-Al_2_O_3_, and c-Y_2_O_3_ remain clearly identifiable, with no abnormal peak splitting or pronounced peak intensification. However, slight shifts are detected in all samples, which may be associated with changes in the residual stress state or local microstructural variations during thermal cycling, but are still insufficient to indicate any pronounced phase transformation. Therefore, under the present experimental conditions, the principal phase constitution of the coatings remains stable before and after thermal cycling, indicating phase transformation is not the dominant factor responsible for coating failure.

**Figure 11:**
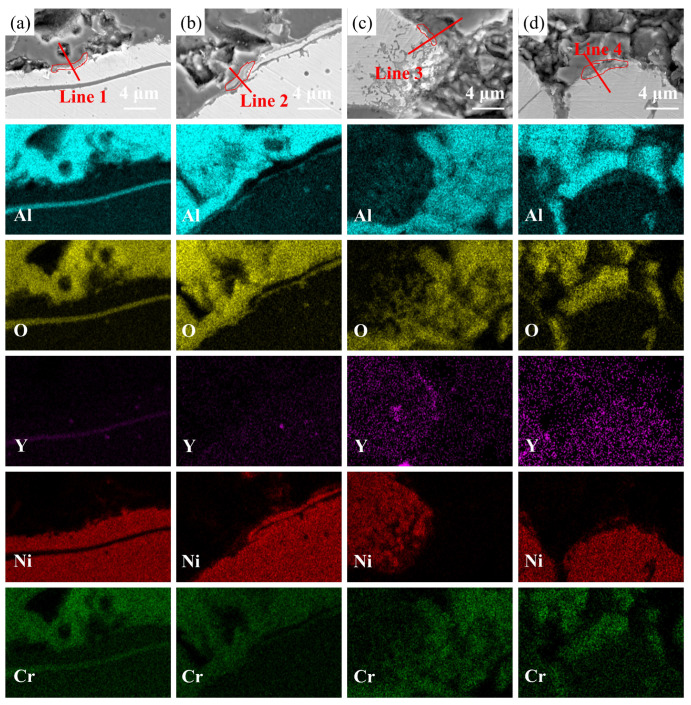
Cross-sectional microstructures and EDS elemental mapping results of the regions adjacent to the TGO after 200 thermal shock cycles: (**a**) A0Y; (**b**) A2Y; (**c**) A5Y; (**d**) A8Y.

**Figure 12:**
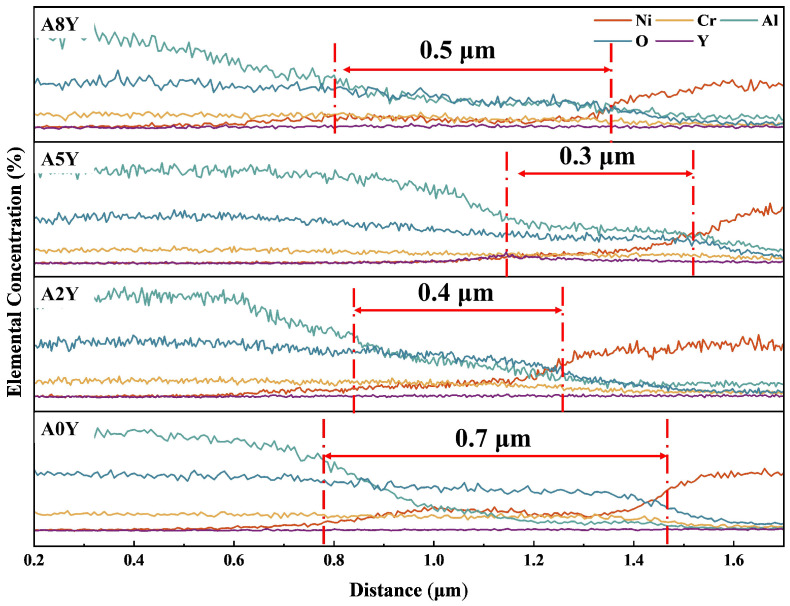
EDS line-scan profiles of Lines 1–4 in Figure 11.

The formation and continued growth of thermally grown oxides (TGO) are widely regarded as important factors contributing to coating failure [27,29,30]. To clarify the formation characteristics and elemental distribution of the TGO layer at the interface between the ceramic top coat and the bond coat after thermal cycling, the interfacial region was characterized by SEM combined with EDS elemental mapping and line scanning, and the results are presented in Figure 11 and Figure 12. The interfacial SEM images reveal a thin and semi-continuous reaction layer between the ceramic top coat and the NiCrAlY bond coat (region marked by the red solid line). This region exhibits a clear contrast difference from the darker Al_2_O_3_ ceramic top coat above and the brighter NiCrAlY bond coat below. The corresponding EDS elemental mapping results show that this layer is distinctly enriched in Al and O, whereas Ni is mainly distributed in the underlying bond coat, indicating that an oxide reaction layer, a TGO layer, forms at the interface after thermal cycling. This layer is primarily composed of Al–O species, with the participation of Cr and Ni [27,29]. Because an Al_2_O_3_ ceramic top coat is used in this study, both the top coat itself and the TGO layer are rich in Al and O. Therefore, it is difficult to accurately distinguish the boundary between the top coat and the interfacial oxide layer solely on the basis of elemental mapping. To address this issue, the line-scan results are further combined to evaluate the evolution of the TGO layer in different coatings after 200 thermal shock cycles. As shown in Figure 11, the Al and O contents remain high within the ceramic top-coat region, whereas the signals of Ni and Cr are close to the background level. In the NiCrAlY bond coat, the contents of Ni and Cr increase markedly, while the O content decreases. Between these two regions, an intermediate zone is observed in which the O content remains high, whereas the Ni and Cr contents are still very low. Therefore, this region can be approximately regarded as the TGO layer formed at the interface. After 200 thermal shock cycles, the TGO layer remains relatively thin overall and exhibits a semi-continuous distribution rather than a fully developed continuous thick oxide scale. The estimated TGO thicknesses of A0Y, A2Y, A5Y, and A8Y are approximately 0.7, 0.4, 0.3, and 0.5 μm, respectively. In other words, the TGO thickness after 200 thermal shock cycles differs significantly among the Al_2_O_3_–Y_2_O_3_ composite coatings with different Y_2_O_3_ contents. These differences in TGO thickness are mainly associated with variations in coating densification, defect connectivity, and interfacial stability [31,32]. Although a TGO layer is observed at the interface of all samples after thermal cycling, its thickness remains significantly lower than the critical value of 5–6 μm reported in the literature for inducing complete delamination of the top coat [26,29]. Therefore, although the slight thickening of the TGO layer may influence the interfacial stress state and local damage evolution, it is not the dominant factor responsible for the thermal cycling failure of the coatings.

During thermal cycling, the ceramic top coat is continuously exposed to a high-temperature environment for prolonged periods and therefore generally undergoes sintering to varying degrees [27]. Previous studies have shown that the healing of interlamellar pores and microcracks during thermal exposure tends to densify the coating microstructure and increase its microhardness and elastic modulus, thereby weakening the ability of the coating to buffer and relax thermal stresses and ultimately impairing its thermal cycling stability [33]. Under the thermal cycling conditions employed in this study, different regions of the ceramic top coat along the thickness direction are exposed to different temperatures, and the internal temperature of the Al_2_O_3_–Y_2_O_3_ composite coating gradually decreases from the surface toward the top coat and bond coat interface.

**Figure 13:**
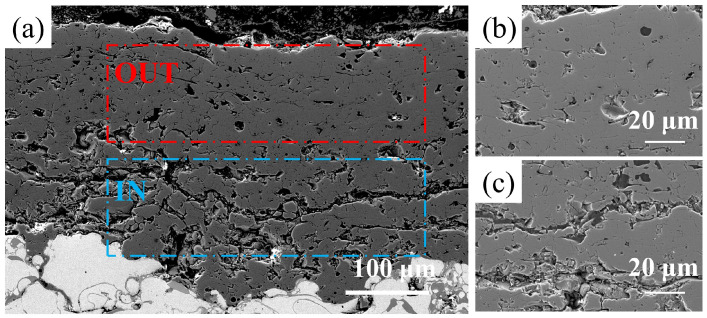
High-magnification SEM images of different regions in the A0Y coating after 200 thermal shock cycles: (**a**) overall cross-sectional SEM morphology; (**b**) SEM morphology of the OUT region; (**c**) SEM morphology of the IN region.

**Figure 14:**
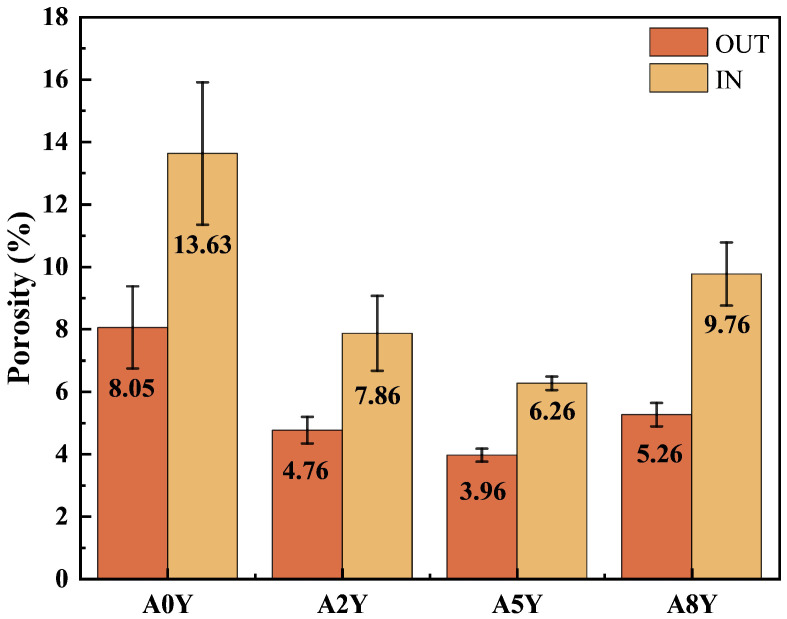
Porosity of different regions along the thickness direction of Al_2_O_3_–Y_2_O_3_ composite coatings.

To elucidate the non-uniform sintering behavior of the ceramic top coat along the thickness direction, the unspalled cross-sectional region of the ceramic top coat is divided into two zones according to its relative position with respect to the thermally grown oxide layer, namely, the “outer layer” (OUT) near the surface and the “inner layer” (IN) adjacent to the interface. Figure 13 shows representative cross-sectional SEM images of the unspalled region after 200 thermal shock cycles, taking the A0Y after 200 cycles as an example. As shown in Figure 13b, in the OUT region of the failed sample, the original lamellar structure becomes markedly less distinct after thermal cycling, and some microcracks exhibit clear signs of healing, indicating pronounced sintering-induced densification in this region. As shown in Figure 13c, because the IN region is exposed to a relatively lower temperature, some lamellar pores are still retained. Meanwhile, owing to the accumulation of thermal-cycling-induced damage, a large number of lamellar pores and cracks are observed in this region. To quantitatively characterize the regional differences in sintering behavior, ImageJ software was used for porosity analysis in different regions of the various samples, and the results are presented in Figure 14. All coatings exhibit a pronounced porosity gradient along the thickness direction after thermal cycling. Specifically, the porosity in the OUT region near the external surface is lower than that in the IN region adjacent to the interface, indicating that the OUT region undergoes more extensive densification during thermal cycling. Combined with Figure 6a and Figure 14, these results confirm that significant non-uniform sintering occurs along the thickness direction of the ceramic top coat during thermal cycling. Meanwhile, clear differences in porosity are still observed among the different samples, indicating that the Y_2_O_3_ content plays an important role in governing the microstructural evolution and sintering behavior of the coatings.

When analyzing crack initiation during thermal-shock cycling, in addition to the factors discussed above, the effects of residual stress and cyclic thermal stress should also be considered. Previous studies have shown that residual stresses in thermally sprayed coatings mainly originate from the quenching stress generated during the rapid solidification of molten droplets and the thermal-mismatch stress formed during the cooling stage. It has been reported that the quenching stress generated during plasma spraying of Al_2_O_3_ coatings is approximately 10 MPa [34,35,36]. However, in the present work, the four coatings were fabricated using the same substrate/bond-coat system, identical spraying parameters, and comparable coating thicknesses. Meanwhile, the thermal expansion coefficients of Al_2_O_3_ and Y_2_O_3_ are very close, i.e., $\alpha_{{Al}_{2}O_{3}}$ = 7.8–8.0 × 10^−6^ K^−1^; $\alpha_{Y_{2}O_{3}}$ = 7.9–8.2 × 10^−6^ K^−1^, and both are much lower than that of the 316L stainless-steel substrate,$\;\alpha_{316L}$ = 19.6 × 10^−6^ K^−1^. Therefore, the residual thermal stresses generated in the four coatings after spraying are expected to be of the same order of magnitude [37,38]. Based on this consideration, the spraying-induced residual stress was not treated as an independent variable in this study. Instead, more attention was paid to the effect of Y_2_O_3_ content on local stress concentration and crack-propagation behavior.

During thermal-shock cycling, repeated heating and water quenching further subject the coating system to significant cyclic thermal stresses. Among these stresses, the thermal-expansion mismatch between the ceramic top coat and the metallic substrate is an important driving force for coating failure. In the present study, although the NiCrAlY bond coat can alleviate the thermal mismatch between the ceramic top coat and the metallic substrate to some extent, Table 4 shows that a pronounced difference in the coefficients of thermal expansion still exists between the ceramic top coat and the 316L stainless-steel substrate. Meanwhile, because the substrate is much thicker than both the bond coat and the ceramic top coat, it exerts a strong constraint on coating deformation during thermal cycling. During heating, thermal mismatch stresses develop in the region adjacent to the interface as a result of the thermal expansion mismatch between the ceramic top coat and the metallic substrate, and local tensile stress zones may form. During the subsequent quenching stage, however, the coating surface cools and contracts rapidly while remaining constrained by the substrate, making the development of relatively high tensile stresses at or near the surface more likely [39]. Therefore, the thermal mismatch strain (*ε*) and thermal mismatch stress (*σ*) generated during thermal cycling can be simply estimated using Equations (4) and (5), respectively [40]:

(4) $$\varepsilon =\left(\alpha_{sub}-\alpha_{Tc}\right)\times \Delta T$$

(5) $$\sigma =E_{Tc}\times \varepsilon =E_{Tc}\times \left(\alpha_{Sub}-\alpha_{Tc}\right)\times \Delta T$$

where Δ*T* is the temperature difference during thermal cycling; α_Tc_ and α_Sub_ are the coefficients of thermal expansion of the ceramic top coat and the substrate, respectively; *E_TC_* is the elastic modulus of the ceramic top coat. By substituting the representative parameters listed in Table 4 into Equation (5), the nominal thermal mismatch stress generated during thermal cycling is estimated to be approximately 0.59 GPa [41]. By contrast, the in-plane stresses induced by sintering are only on the order of several tens of MPa. In addition, previous studies on thermal barrier coatings (TBCs) have shown that failure caused by thermal expansion mismatch during thermal cycling predominantly manifests as interfacial delamination, which is highly consistent with the failure mode observed in the present study [42,43,44]. Therefore, the cyclic thermal stresses induced by thermal expansion mismatch can be regarded as the dominant driving force for crack initiation, propagation, and ultimate coating failure, whereas sintering is more likely to accelerate the failure process by increasing the elastic modulus and reducing the strain tolerance of the coating. During repeated thermal cycling, thermal stresses progressively accumulate and preferentially induce horizontal cracks at the interface between the ceramic layer and the bond coat (Figure 9b), thereby exerting a detrimental effect on the thermal shock resistance of the coating [24,44]. As thermal cycling proceeds, vertical cracks gradually propagate toward the interface and interact with the pre-existing horizontal interfacial cracks, thereby promoting crack extension along the ceramic top coat and bond coat interface and ultimately leading to coating spallation.

Based on the above analysis, the addition of Y_2_O_3_ affects the thermal shock resistance of the coatings, leading to the following order for the Al_2_O_3_–Y_2_O_3_ composite coatings: A5Y > A2Y > A8Y > A0Y. The primary reason is that the Y_2_O_3_ content alters the initial microstructure of the coatings, which in turn affects their resistance to crack initiation and propagation. Although A0Y is characterized by relatively small pore sizes, it exhibits a comparatively high porosity, and many of these pores are irregularly shaped defects formed by insufficient spreading and overlapping of particles during the APS process. Such defects cannot effectively relieve coating stresses and instead tend to act as weak sites for crack initiation and propagation [46]. When the Y_2_O_3_ content increases to 2–5 wt.%, the coating porosity decreases and the microstructural uniformity improves, which enhances the coating microhardness and crack resistance, suppresses crack initiation and propagation within the coating, and thereby improves the thermal shock resistance of the Al_2_O_3_–Y_2_O_3_ composite coatings. Previous studies have likewise confirmed that an appropriate amount of porosity can be beneficial to thermal shock resistance [47]. However, when the Y_2_O_3_ content is further increased to 8 wt.%, the number of pores in the coating rises again, and some pores become elongated and relatively large. Previous studies have shown that such large pores readily act as stress concentration sites under thermal stress and promote the preferential initiation of cracks [20,48]. In addition to internal pore defects, the higher surface roughness of A8Y may also contribute to local stress concentration during thermal shock cycling. As shown in Figure 4e, A8Y exhibits the highest surface roughness, indicating the presence of more semi-molten or insufficiently melted particles on the coating surface. These surface asperities can introduce local geometric discontinuities and cause non-uniform thermal contraction during rapid cooling, leading to stress concentration around asperity roots, surface-connected pores, and microcracks [49]. Consequently, the reduced thermal shock resistance of A8Y is related to the combined effects of large pores, high surface roughness, and increased microstructural heterogeneity.

## 4. Conclusions

In this study, Al_2_O_3_–Y_2_O_3_ composite coatings with different Y_2_O_3_ contents were prepared by APS, and their phase constitution, microstructure, mechanical properties, and thermal shock resistance were systematically investigated. The main conclusions are as follows:

1. The Al_2_O_3_–Y_2_O_3_ composite coatings fabricated by APS are mainly composed of γ-Al_2_O_3_, α-Al_2_O3, c-Y_2_O_3_, and a small amount of m-Y_2_O_3_. With increasing Y_2_O_3_ content, the intensity ratio of α-Al_2_O_3_ (113) to γ-Al_2_O_3_ (440) increases from 0.31 for A0Y to 0.86 for A8Y, indicating that the addition of Y_2_O_3_ exerts a significant stabilizing effect on the α-Al_2_O_3_ phase.
2. Y_2_O_3_ content has a pronounced influence on the as-sprayed microstructure and defect characteristics of the composite coatings. A2Y and A5Y show improved deposition states and reduced porosities of 5.70 ± 0.54% and 4.72 ± 0.28%, respectively, whereas A8Y exhibits an increased porosity of 7.13 ± 0.59% due to more insufficiently melted particles. The pore structure also changes from mainly small pores of 0–50 μm^2^ in A0Y to more large pores of 100–300 μm^2^ in A8Y.
3. The mechanical properties are closely related to the coating densification and defect characteristics. Compared with A0Y, which exhibits a microhardness of 548.8 ± 33.1 HV_0.5_ and an indentation fracture toughness of 0.96 ± 0.25 MPa·m^1/2^, A2Y and A5Y show improved properties, reaching 708.1 ± 48.4 HV_0.5_ and 1.71 ± 0.31 MPa·m^1/2^ for A2Y, and 686.6 ± 45.6 HV_0.5_ and 1.69 ± 0.40 MPa·m^1/2^ for A5Y. In contrast, A8Y decreases to 445.4 ± 73.9 HV_0.5_ and 1.38 ± 0.37 MPa·m^1/2^ due to increased large pores, unmelted/semi-molten particles, and microstructural heterogeneity.
4. The addition of an appropriate amount of Y_2_O_3_ significantly improves the thermal shock resistance of Al_2_O_3_-based coatings. After 200 water-quenching thermal shock cycles at 600 °C, the thermal shock resistance follows the order A5Y > A2Y > A8Y > A0Y. A5Y shows the best performance, with a spallation area ratio of only 0.29% and a mass loss of 1.5 mg·cm^−2^, whereas A0Y suffers the most severe damage, with a spallation area ratio of 15.17% and a mass loss of 7.5 mg·cm^−2^.
5. The thermal cycling failure of the Al_2_O_3_–Y_2_O_3_ composite coatings is mainly driven by cyclic thermal stresses induced by thermal expansion mismatch, which ultimately leads to localized spallation through the initiation, accumulation, and propagation of cracks associated with pores, lamellar boundaries, and regions adjacent to the interface. An appropriate Y_2_O_3_ addition optimizes the coating microstructure and enhances crack propagation resistance, thereby effectively improving thermal shock resistance. In contrast, excessive Y_2_O_3_ addition weakens this beneficial effect because of increased microstructural heterogeneity and the higher density of defects.

## Figures and Tables

**Table 1:** Spraying parameters for the fabrication of the NiCrAlY bond coat and Al_2_O_3_ ceramic top coat by atmospheric plasma spraying.

Parameters	Arc Voltage, V	Arc Current, A	Ar Flow Rate, L/min	H_2_ Flow Rate, L/min	Carrier Gas Flow Rate, L/min	Spray Distance, mm	Powder Feed Rate, g/min	Number of Spraying Passes
Al_2_O_3_	72	560	40	10	4	160	35	25–28
Al_2_O_3_ + Y_2_O_3_	72	560	40	10	4	160	35	25–28
NiCrAlY	70	520	50	10	4	160	25	4–5

**Table 2:** Intensity ratios of the characteristic peaks corresponding to the (113) plane of α-Al_2_O_3_ and the (440) plane of γ-Al_2_O_3_.

Specimen	(113) Diffraction Peak Intensity	(440) Diffraction Peak Intensity	I(113)/I(440)
A0Y	680	2146	0.31
A2Y	705	1657	0.42
A5Y	727	1124	0.65
A8Y	877	1020	0.86

**Table 3:** Mechanical properties of the Al_2_O_3_–Y_2_O_3_ composite coatings.

Specimen	Feedstock Composition	Microhardness (HV_0.5_)	Fracture Toughness (MPa·m^1/2^)
A0Y	Al_2_O_3_	548.8 ± 33.1	0.96 ± 0.25
A2Y	Al_2_O_3_ + 2 wt.%Y_2_O_3_	708.1 ± 48.4	1.71 ± 0.31
A5Y	Al_2_O_3_ + 5 wt.%Y_2_O_3_	686.6 ± 45.6	1.69 ± 0.40
A8Y	Al_2_O_3_ + 8 wt.%Y_2_O_3_	445.4 ± 73.9	1.38 ± 0.37

**Table 4:** Representative parameters used for estimating the thermal mismatch stress.

Parameter		Value Used
Thermal expansion coefficient (K^−1^) [30]	316L	$\alpha_{Sub}$ = 18.2 × 10^−6^
	Al_2_O_3_/Al_2_O_3_-Y_2_O_3_	$\alpha_{TC}$ = 11.3 × 10^−6^
	NiCrAlY	$\alpha_{BC}$ = 8.0 × 10^−6^
Effective elastic modulus of APS ceramic top coat (GPa)		*E_TC_* = 100
Temperature difference (K)		ΔT = 580

(*E_TC_* = 100 GPa was selected as a representative value based on the experimentally measured elastic modulus ranges reported for plasma-sprayed alumina coatings, namely 77–135 GPa [44] and 110 ± 40 GPa [45]).

## Data Availability

The original contributions presented in this study are included in the article. Further inquiries can be directed to the corresponding authors.
